# Fentanyl versus placebo with ketamine and rocuronium for patients undergoing rapid sequence intubation in the emergency department: The FAKT study—A randomized clinical trial

**DOI:** 10.1111/acem.14446

**Published:** 2022-03-15

**Authors:** Ian Ferguson, Alexander Buttfield, Brian Burns, Cliff Reid, Shamus Shepherd, James Milligan, Ian A. Harris, Anders Aneman, the Australasian College for Emergency Medicine Clinical Trials Network

**Affiliations:** ^1^ South West Clinical School University of New South Wales Sydney New South Wales Australia; ^2^ Emergency Department Liverpool Hospital Sydney New South Wales Australia; ^3^ GSA‐HEMS, NSW Ambulance Bankstown Aerodrome Sydney New South Wales Australia; ^4^ University of Western Sydney Sydney New South Wales Australia; ^5^ Campbelltown Hospital Sydney New South Wales Australia; ^6^ University of Sydney, Discipline of Emergency Medicine Sydney New South Wales Australia; ^7^ Northern Beaches Hospital Sydney New South Wales Australia; ^8^ Orange Health Service Orange New South Wales Australia; ^9^ University of New South Wales Rural Clinical School Orange New South Wales Australia; ^10^ Royal North Shore Hospital, St Leonards Sydney New South Wales Australia; ^11^ CareFlight Ltd Sydney New South Wales Australia; ^12^ Ingham Institute for Applied Medical Research Liverpool New South Wales Australia; ^13^ Intensive Care Unit, Liverpool Hospital Liverpool New South Wales Australia

## Abstract

**Objective:**

The objective was to determine whether the use of fentanyl with ketamine for emergency department (ED) rapid sequence intubation (RSI) results in fewer patients with systolic blood pressure (SBP) measurements outside the pre‐specified target range of 100–150 mm Hg following the induction of anesthesia.

Methods

This study was conducted in the ED of five Australian hospitals. A total of 290 participants were randomized to receive either fentanyl or 0.9% saline (placebo) in combination with ketamine and rocuronium, according to a weight‐based dosing schedule. The primary outcome was the proportion of patients in each group with at least one SBP measurement outside the prespecified range of 100–150 mm Hg (with adjustment for baseline abnormality). Secondary outcomes included first‐pass intubation success, hypotension, hypertension and hypoxia, mortality, and ventilator‐free days 30 days following enrollment.

**Results:**

A total of 142 in the fentanyl group and 148 in the placebo group commenced the protocol. A total of 66% of patients receiving fentanyl and 65% of patients receiving placebo met the primary outcome (difference = 1%, 95% CI = −10 to 12). Hypotension (SBP ≤ 99 mm Hg) was more common with fentanyl (29% vs. 16%; difference = 13%, 95% CI = 3% to 23%), while hypertension (≥150 mm Hg) occurred more with placebo (69% vs. 55%; difference = 14%, 95% CI = 3 to 24). First‐pass success rate, 30 day mortality, and ventilator‐free days were similar.

**Conclusions and Relevance:**

There was no difference in the primary outcome between groups, although lower blood pressures were more common with fentanyl. Clinicians should consider baseline hemodynamics and postinduction targets when deciding whether to use fentanyl as a coinduction agent with ketamine.

## INTRODUCTION

Rapid sequence intubation (RSI) in the emergency department (ED) occurs frequently in patients with pathology that is undifferentiated at the time of the procedure. Because significant hemodynamic fluctuations may worsen patient outcomes, notably in conditions such as head injury, myocardial ischaemia, or aneurysmal subarachnoid hemorrhage, induction medications that reduce the incidence of both hypotension and hypertension are preferred.

Etomidate has frequently been the induction agent of choice in these circumstances but is not universally available internationally, and concerns regarding adrenal suppression have also limited its use. Previous recommendations against the use of ketamine have been challenged^1^ and a recent study has demonstrated an increase in its use.^2^

Ketamine is less likely to cause hypotension than most other induction agents,^3^ but tachycardia and hypertension are frequently observed that may in themselves worsen patient outcomes, particularly in patients with conditions such as vasculopathies or intracranial or other critical bleeding. Fentanyl may be used as an adjunctive induction medication with ketamine to moderate hypertension following the induction of anesthesia, but it may increase the risk of hypotension, which is associated with increased mortality rates.^4^ It is also unclear whether the addition of fentanyl affects intubating conditions or patient‐centered outcomes such as mortality. A recent systematic review only identified one relevant trial, which was an observational study that demonstrated a risk of postinduction hypotension with fentanyl.^5^

Clinicians need a clear understanding of the hemodynamic effects of ketamine with and without fentanyl in ED patients who require RSI, and as such we aimed to investigate whether the addition of fentanyl to a standardized anesthetic induction regimen of ketamine and rocuronium in adult patients undergoing RSI in the ED improved postinduction hemodynamic stability. The secondary aims were to investigate any effect on intubating conditions and 30‐day mortality.

## METHODS

### Study design

We conducted a multicenter, randomized double‐blind placebo‐controlled trial comparing fentanyl versus placebo, in addition to ketamine and rocuronium, according to a weight‐based dosing schedule for RSI in adult ED patients. The protocol was approved by the South West Sydney Local Health District Human Research and Ethics Committee (HREC/17/LPOOL/450) with a waiver of informed consent. The protocol and analysis plan were published prior to completion of recruitment.^6^ The trial is registered with the Australian and New Zealand Clinical Trials Registry (ACTRN12616001570471).

### Study setting and participants

Study sites were the EDs of five hospitals in New South Wales, Australia: Liverpool Hospital (adult and pediatric academic center, annual ED census of 100,000 patients); Campbelltown Hospital (adult and pediatric community hospital, annual ED census 85,000 patients); Northern Beaches Hospital (adult and pediatric community hospital, annual census 70,000 patients); Orange Health Service (adult and pediatric rural community hospital, annual census 31,000); and Royal North Shore Hospital (adult and pediatric academic center, annual ED census 95,000 patients). Adult patients (≥18 years) who required RSI were screened for eligibility. Exclusion occurred due to allergy to study medication, the need for a "paralysis‐only" or no‐drug intubation, an alternative induction regime being necessary, the ED being overwhelmed (both in the opinion of the treating emergency physician) or because no specialist trained in the protocol was available.

### Interventions

Prefilled syringes containing either 200 μg fentanyl in 20 ml (intervention) or 20 ml of 0.9% saline (control) were prepared by a third‐party compounding service (Baxter Pharmaceutical) in batches of eight, with each batch consisting of four fentanyl and four placebo syringes. Batches of eight syringes were distributed to participating hospital pharmacies, where an unblinded pharmacist labeled each syringe with a sequential unique study number generated by a block randomization schedule, before delivery to the ED. Participants were randomized to receive either fentanyl or placebo in a 1:1 ratio with participants and clinical and research staff blinded to the study allocation throughout the duration of the study. The data analyses were performed with the study groups identified as “1” or “2” and the manuscript was written and approved by all authors before the study randomization code was revealed.

Patients were prepared for intubation in a standardized manner, including:

1. Monitoring (noninvasive blood pressure at 2‐min intervals and continuous electrocardiographic, oxygen saturation, and end‐tidal carbon dioxide monitoring);
2. Resuscitation, with optimization of preoxygenation (including noninvasive ventilation if necessary) and hemodynamic status (including intravenous volume expansion and inotropic medications where needed);
3. Position optimization; and
4. Completion of an airway checklist.

No specific end points for resuscitation were used, and the timing of proceeding with RSI following optimization was at the discretion of the treating emergency physician, in keeping with normal clinical practice.

Physicians determined the study drug volume administered by calculating the intended ketamine dose (doses of 1–2 mg/kg ketamine for standard dosing and 0.5–1 mg/kg for reduced dosing, using a 200 mg/20 ml formulation) and then matching this with the volume of study drug (i.e., a patient receiving 100 mg (10 ml) of ketamine would also receive 10 ml of study drug (either 100 μg fentanyl or 10 ml of 0.9% saline). This 1:1 ratio of fentanyl to ketamine was determined by pragmatic consensus among the author group.

Study medications were administered in the order of study drug, *then* ketamine, and *then* rocuronium in a rapid sequential fashion. Sixty seconds following the completion of the rocuronium bolus, the first attempt at laryngoscopy could commence. Aspects of the intubation procedure following this were recorded but remained at the discretion of the treating team. Additional sedative medication was discouraged until 10 min following induction, although patient care was prioritized over research procedures in the event that these conflicted. Sedation was continued after this interval using intravenous fentanyl and propofol.

### Measurements

Baseline characteristics were recorded, with physiologic variables drawn from monitoring including continuous three‐lead electrocardiography and pulse oximetry, noninvasive blood pressure, and end‐tidal capnography. Incomplete information with regard to baseline characteristics (for example, due to an unidentified patient or incomplete medical history at the time of arrival) were subsequently sought from the electronic medical record by a member of the research team.

Drug dosing was determined using a standardized weight‐based dosing table (Table 2; Supplement 1), allowing for both standard and reduced dosing of sedative medications at the discretion of the treating clinician. The rocuronium dosing remained the same (1.5 mg/kg) regardless of patient stability.

Following induction, pulse, noninvasive blood pressure, oxygen saturations, and end‐tidal carbon dioxide levels were recorded every 2 min by a nominated member of the clinical team. Suspected erroneous measurements in the judgment of the treating team were repeated after adjustment of monitoring.

The type of laryngoscope, grade of view, use of bougie or stylet, need for manual in‐line stabilization, and number of intubation attempts were recorded contemporaneously, as were immediate complications, including esophageal intubation and need for rescue airway intervention.

Main final diagnosis, mortality status and number of ventilator‐free calendar days at 30 days following intubation were collected after 30 days by a member of the research team at each of the participating hospitals, by interrogation of the electronic medical record. Study data were collected and managed using REDCap (Research Electronic Data Capture) electronic data capture tools hosted at the University of New South Wales.^7^, ^8^ Data collected at the time of intubation were either entered onto a paper case report form and then entered into REDCap by the research team or entered directly into REDCap. Follow‐up data were entered directly into REDCap by the research team.

### Outcomes

The determination of the primary outcome was challenging, due to there being no consistent approach in the literature. The approach taken by Lyon et al.^9^ in their before‐and‐after study comparing two induction regimes of using a change of 20% from baseline blood pressure was felt to be inappropriate for an ED study due to the potential for significant physiological disruption at baseline. Jabre et al.^11^ in a randomized controlled trial comparing ketamine with etomidate for prehospital RSI used the sequential organ failure assessment (SOFA) score, which is a predictor of intensive care unit mortality as a primary outcome, but this may be confounded by downstream treatments and can be subject to data entry errors. Given the potential drawbacks of these methods, by pragmatic consensus among the author group, the primary outcome was to be met if the systolic blood pressure (SBP) fell outside the limits of 100–150 mm Hg at any of the 2‐min time points up until 10 min following the commencement of the study drug bolus. Patients with a SBP ≥ 151 mm Hg prior to induction met the primary outcome if their SBP rose by ≥10% or fell outside the lower limit at any of the time points postinduction, whereas patients whose SBP at induction was ≤ 99 mm Hg met the primary outcome if their postinduction SBP fell by ≥10% or fell outside the upper limit during this period.

These limits of normality were chosen as there is evidence for using thresholds between 90 and 110 mm Hg for hypotension^12^, ^13^ and between 140 and 160 mm Hg for hypertension^14^, ^15^ in acute emergent pathologies, and as such in undifferentiated patients it was felt that these were levels that emergency physicians would typically target.

Secondary outcomes of hypoxia (SpO_2_ ≤ 93%), tachycardia (HR ≥ 120), and cardiac arrest occurring within 10 min of induction were recorded, as were the laryngoscopic view, first‐pass intubation success rate, use of supraglottic airway devices, and need for a surgical airway. Mortality and number of ventilator‐free calendar days were both recorded at 30 days, when the final diagnosis was also recorded. It was prospectively decided that patients discharged from hospital at their baseline level of function before 30 days were deemed to be alive at 30 days.

### Data analysis

Prior study suggested that approximately 80% of the control group and 40% of the intervention would meet the primary endpoint^9^; however, because this was based on retrospective observational data, we theorized that the actual difference would be smaller and therefore rates of 70% and 50% were used to reduce the likelihood of type II error. An absolute risk reduction for the primary outcome of 20%, i.e., a number needed to treat of 5, was considered to represent a clinically important difference. At a power of 90% and a two‐sided significance level set at 0.05, a sample size of 134 in each group was required; a total of 300 participants (150 in each arm) were chosen to allow for a study attrition rate of 10%.

A single, planned interim analysis was conducted by the Data Safety and Monitoring Committee (DSMC) with data from the first 150 participants using prespecified stopping criteria for safety (excess mortality or hypotension in either arm, at the p < 0.001 level). The primary analysis was an unadjusted, modified intention‐to‐treat comparison of the proportion of patients in each group meeting the primary outcome using a chi‐square test. Categorical outcomes are presented as numbers and percentages, with absolute risk differences presented as percentages with 95% confidence intervals (CIs). Between‐group differences for repeated measurements over time were assessed using a two‐way analysis of variance having ensured that the assumptions regarding outliers, normality of distribution of the dependent variable (using the Shapiro‐Wilks test), and homogeneity of variance (using Levine's test) were all met. Due to missing data post hoc sensitivity analyses were conducted assuming that all patients for whom primary outcome data was missing either met or did not meet the primary outcome. The type I error rate was set at the level of 0.05. Analyses were conducted using STATA version 15.0 (Statacorp).

## RESULTS

### Characteristics of study subjects

**FIGURE 1:**
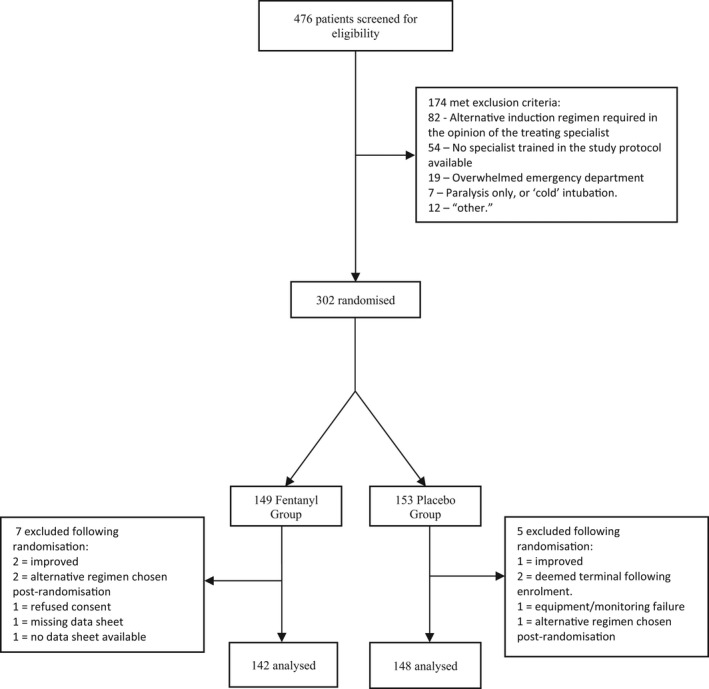
Study flow (CONSORT) diagram

Eligible patients were recruited between August 25, 2018, and December 3, 2020. Of 474 patients screened for inclusion, 302 were enrolled and randomly assigned to receive either fentanyl or placebo. A total of 143 patients in the fentanyl group and 148 patients in the placebo group entered the clinical protocol (see Figure 1). In the fentanyl group one patient withdrew consent after being included in the study, and their data were withdrawn. Follow‐up data were available on all of the remaining 277 patients who were included in a modified intention‐to‐treat analysis. Of the included patients, 190 were recruited in tertiary care centers, 91 were recruited in urban district hospitals and nine were recruited in the rural base hospital. There was no stratification by hospital site.

### Results

Baseline characteristics were similar between both groups and are listed in Table 1. Primary outcome data were available for 277 of 290 (95.5%) participants (Table 2). In the fentanyl group 66% of patients met the primary outcome versus 65% in the placebo group (difference = 1%, 95% CI = −10% to 12%, p = 0.86). In the remaining 13 patients, there were insufficient data to determine whether the primary outcome had been met, and so sensitivity analyses assuming that all of the missing patients either (1) met or (2) did not meet the primary outcome were conducted. These showed no statistical difference between the groups (differences = [1] 1%, 95% CI = −10% to 12%; [2] 5%, 95% CI = −6% to 16%).

**TABLE 1:** Baseline characteristics

	Fentanyl (*n* = 143)	Placebo (*n* = 148)
Age (years)	55 [39–71]	54 [36–67]
Sex		
Male	92 (65)	78 (53)
Female	35 (35)	70 (47)
Estimated weight (kg)	80 [70–90]	77.5 [65–90]
Comorbidities		
Hypertension	40 (29)	41(28)
Diabetes mellitus	22 (15)	25 (17)
COPD/asthma	18 (7)	16 (11)
Epilepsy	14 (10)	12 (8)
Ischemic heart disease	20 (14)	14 (9)
Cerebrovascular disease	11 (8)	5 (3)
Cancer (active)	6 (4)	3 (2)
Medications		
Aspirin	19 (13)	14 (9)
Clopidogrel	10 (7)	3 (2)
Other antiplatelet	2 (1)	0 (0)
Warfarin	7 (5)	5 (3)
DOAC	12 (8)	7 (5)
NSAID	0 (0)	2 (1)
Beta‐blocker	19 (13)	15 (10)
ACE‐inhibitor/ARB	16 (11)	18 (12)
Other antihypertensive	20 (14)	17 (11)
Other medications	71 (50)	74 (50)
No medications	26 (18)	27 (18)
Unknown medications	21 (15)	36 (24)
Team Leader		
Emergency medicine specialist	110 (77)	120 (81)
Emergency medicine registrar	33 (23)	28 (19)
Indication for intubation medical:	128 (90)	127 (86)
Overdose	52 (36)	52 (35)
Stroke/intracranial hemorrhage	17 (12)	19 (13)
Seizure	17 (12)	15 (10)
Altered level of consciousness (medical etiology)	13 (9)	17 (11)
Respiratory failure	12 (8)	9 (6)
Post–cardiac arrest	8 (6)	9 (6)
Cardiac failure	3 (2)	2 (1)
Sepsis	3 (2)	2 (1)
Gastrointestinal bleed	1 (1)	0 (0)
Other	2 (1)	2 (1)
Trauma	15 (10)	21 (14)
Reduced level of consciousness	11 (7)	12 (8)
Neck/facial trauma	1 (1)	4 (3)
Chest trauma	0 (0)	4 (3)
Traumatic cardiac arrest	2 (1)	0 (0)
Shock	0 (0)	1 (1)
Burn/inhalation	1 (1)	0 (0)
Preintubation resuscitation		
Vasopressor use at time of intubation	17 (12)	18 (12)
Crystalloid volume administered prior to induction (mL), median (IQR) [range]	500 (250–1000) [250–4500]	500 (250–1000) [250–5000]
Baseline vital signs		
SBP (mm Hg)	132 (112–149)	135 (116–155)
SBP ≤ 99 mm Hg at baseline	10 (7)	10 (7)
SBP ≥ 151 mm Hg at baseline	34 (24)	44 (30)
Glasgow Coma Scale score	5 (3–8)	6 (3–9)
Respiratory rate	18 (14–21)	19 (15–22)
Pulse, mean (95% CI)	92 (88–97)	96 (92–100)
Oxygen saturation, median (IQR) [range]	100 (98–100) [83–100]	100 (98–100) [84–100]
Induction medication		
Ketamine dose (mg/kg)	1.4 (1–1.5)	1.25 (1–1.5)
Study drug volume (ml/kg)	0.14 (0.1–0.15)	0.13 (0.1–0.15)
Rocuronium dose (mg/kg)	1.55 (1.5–1.7)	1.55 (1.5–1.7)

*Note:* Data are reported as median (IQR) or *n* (%), unless otherwise indicated.

Abbreviations: COPD, chronic obstructive pulmonary disease; NSAID, nonsteroidal anti‐inflammatory drug.

**TABLE 2:** Primary and secondary endpoints

	Fentanyl	Placebo	Difference	*p*‐value
Primary endpoint (SBP outside target range^a^ within 10 min of induction)	92/140 (66)	89/137 (65)	1 (−10 to 12)	0·86
Hypotension (SBP < 100 mm Hg within 10 min of induction)^b^	42 (29)	24 (16)	13 (3 to 23)	
Hypertension (SBP > 150 mm Hg within 10 min of induction)^b^	79 (55)	102 (69)	14 (3 to 24)	
Tachycardia (HR ≥ 120 within 10 min of induction)	68 (48)	91 (61)	13 (2–25)	
Hypoxia (SpO_2_ < 93% within 10 min of induction)	28 (19)	19 (13)	6 (−2 to 15)	
Cardiac arrest	2 (1.4)	1 (0.7)	1 (−1 to 3.0)	
30‐day mortality	27 (19)	35 (24)	5 (−4 to 15)	
Ventilator‐free days, median (IQR)	28 (27–29)	28 (26–29)	0 (−6 to 6)	
First‐pass success	132 (92)	136 (92)	0 (−6 to 0.6)	
Cormack and Lehane view on first‐attempt laryngoscopy				
Grade I	87 (61)	92 (62)	1 (−12 to 10)	
Grade II	42 (29)	43 (29)	0 (−10 to 10)	
Grade III	11 (7)	7 (5)	2 (−3 to 7)	
Grade IV	3 (2)	5 (3)	1 (0 to 2)	
Use of supraglottic rescue device	0 (0)	2 (1)	1 (−1 to 3)	
Need for surgical airway (%)	0 (0)	(0)	0 (0)	
Patients included in subgroup receiving < 1 mg/kg ketamine	56 (39)	49 (33)	6 (−5 to 17)	
Patients in subgroup meeting primary outcome	34 (61)	31 (63)	2 (−16 to 20)	

*Note:* Data are reported as n (%) or percent (95% CI), unless otherwise noted. Data available for SBP at 1334 of a potential 1450 (92%) time points following induction.

Abbreviations: HR, heart rate; SBP, systolic blood pressure; SpO_2_, oxygen saturation.

^a^ Target range = 100–150 mm Hg or a change of ≤10% from baseline in a congruent direction if baseline SBP outside 100–150 mm Hg.

^b^ Regardless of baseline SBP.

Despite the similarities in the primary outcome, the distribution of blood pressure between the two groups was different, with 29% of patients in the fentanyl group having at least one SBP measurement of less than 100 mm Hg compared to 16% of the placebo group (difference = 13%, 95% CI = 3% to 23%) and with 69% of the placebo group having a SBP measurement of more than 150 mm Hg compared to 55% of the fentanyl group (difference = 14%, 95% CI = 3% to 24%). Secondary outcomes are shown in Table 2, with more tachycardia in the placebo group (48% vs. 61%: difference = 13%, 95% CI = 2% to 25%). The incidence of hypoxia was 13% in the placebo group versus 19% in the fentanyl group (difference = 6%, 95% CI = −2% to 15%). Procedural secondary outcomes were similar between groups, including laryngoscopic view, first‐pass intubation rate, and need for rescue airway techniques (Table 2). The 30‐day mortality was 19% in the fentanyl group 24% in the placebo group (difference = 5%, 95% CI = −4% to 15%). The median number of ventilator‐free calendar days was the same in both groups.

**FIGURE 2:**
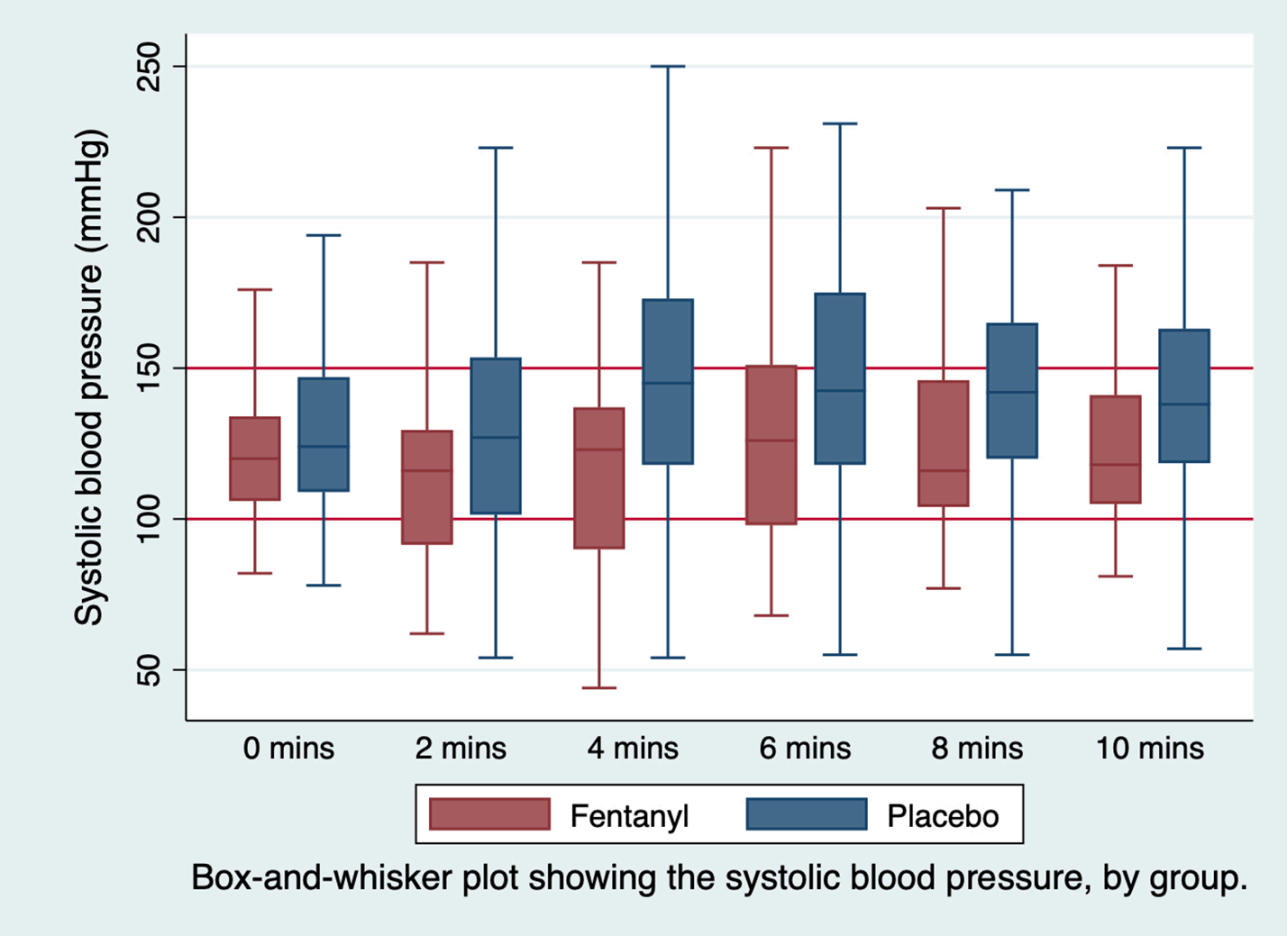
Box‐and‐whisker plot showing systolic blood pressure at induction each 2‐minute time point until 10‐minutes, by group

**FIGURE 3:**
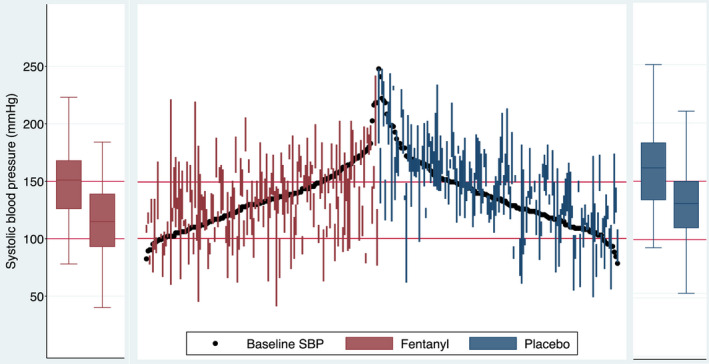
Vertical line plot showing the baseline systolic blood pressure for individual patients (solid black dots), with corresponding vertical lines representing the minimum and maximum systolic blood pressure measured in each individual patients during the ten minutes following induction. The box‐and‐whisker plots flanking the main plot demonstrate the minimum (right plot) and maximum (left plot) systolic blood pressure for each treatment group during the ten minutes following induction

Figure 2 demonstrates the lower median blood pressures in the fentanyl group at each of the 2‐min time points following the induction of anesthesia, while Figure 3 shows the baseline blood pressure as well as the maximum and minimum blood pressure (denoted by the extremes of each vertical line) for each individual patient. The box‐and‐whisker plots represent the maximum and minimum blood pressure for each group during the 10 min following induction. This demonstrates graphically that hypertension is commoner with placebo, while hypotension is seen more commonly with fentanyl use.

A preplanned subgroup analysis of participants receiving <1 mg/kg ketamine included 49 patients (33%) from the placebo group and 56 (39%) from the fentanyl group (difference = 6%, 95% CI = −5% to 17%; p = 0.28). The primary outcome was met in 31 participants (63%) in the placebo group, compared to 34 participants (61%) in the fentanyl group, an absolute difference of 2% (95% CI = −15% to 19%; p = 0.82).

## DISCUSSION

In this randomized placebo‐controlled trial of ketamine with or without the use of fentanyl for ED RSI, no difference was seen in the proportion of patients in each group who had at least one SBP measurement outside the target range of 100–150 mm Hg during the 10 min following induction, although the distribution of blood pressures between groups is demonstrably different, with lower blood pressures being seen with the addition of fentanyl to the regimen. While we acknowledge that this primary end point is not truly patient‐centred, we would assert that the difference seen between groups is of interest and relevance to clinicians who undertake intubation within the ED.

While less than 1% of patients presenting to the ED require RSI,^16^ it is a critical procedure with potential for significant morbidity. The diagnosis at the point of anesthesia is frequently unclear but the assumption that there may be a pathological process under way, which may be critically influenced by hemodynamic changes is reasonable. A strategy aiming to normalize postinduction hemodynamics seems warranted in the face of currently available evidence, although the parameters that define “normal” are not well defined in the literature.^12^, ^13^, ^14^, ^15^ The proportion of patients who maintained SBP within a 100–150 mm Hg range was almost identical in each group, but the distribution of blood pressures was different. An increased incidence of hypotension was observed in the fentanyl group and increased hypertension in the placebo group. The addition of fentanyl to an induction regimen of ketamine and rocuronium thus altered the postinduction hemodynamic profile at each of the recorded time points.

Previous literature on this subject has been conflicting. Lyon et al.^9^ demonstrated an amelioration of the hypertensive effects of ketamine with fentanyl, without increased hypotension, although missing data were a significant limitation of their study. They also conducted their study in a population of prehospital trauma patients, compared to our more heterogenous patient group. Takahashi et al.^17^ showed an association with increased rates of hypotension when fentanyl was used as an adjunct, although these cases included multiple sedative agents and relatively few instances of ketamine use. Miller et al.^18^ specifically considered the hemodynamics of RSI with ketamine in a cohort where fentanyl pretreatment was uncommon (fentanyl was used in approximately 10% of cases), comparing patients with or without shock. A significant increase in SBP was observed in the unshocked group, whereas most patients who were shocked did not see this change; further study on the effects of fentanyl in unshocked patients receiving ketamine for anesthetic induction was recommended.

It is generally accepted that postintubation hypotension in susceptible patients results in increased mortality,^3^, ^12^ but the effect of hypertension is less clear. A retrospective study considering prehospital RSI in stroke demonstrated an association between hypertension and increased mortality,^19^ with the authors speculating that hypertension may be associated with increased bleeding and cerebral edema. Postintubation hypertension has been demonstrated at rates varying from 9% to 80% in the prehospital and ED setting,^9^, ^10^, ^19^, ^20^ and there are potential mechanisms by which it could impact on mortality including increased bleeding, myocardial ischemia, and aneurysmal rupture. Hypertension was the more frequent observation in our study in both groups, but adjunctive fentanyl blunted the hypertensive response to RSI with ketamine, although at the price of more hypotension.

A statistically significant difference in heart rate was observed between the fentanyl and placebo groups. While there is some evidence that controlling tachycardia in some conditions such as aortic dissection^21^ and aneurysmal subarachnoid hemorrhage^22^ may improve outcomes, the magnitude of the difference in heart rates seen in our study was small and so the clinical significance of this is likely to be negligible. The incidence of cardiac arrest was similar in both arms and appears to be consistent with other literature.^23^, ^24^ It is important to note that this study was not powered to detect a difference in mortality. Despite prior data on the impact of postintubation hypotension^3^, ^12^ no difference in mortality was demonstrated between the groups studied, although the rate of hypotension was significantly greater with fentanyl.

The first‐pass intubation success rates were high in both groups and all patients were intubated successfully within three attempts, with low rates of rescue intervention and no need for surgical airways. This suggests that the addition of fentanyl had no impact on intubation conditions or intubation success rates when an adequate dose of paralysis medication is used. Overall high first‐pass success rate in this cohort is a reasonable reflection of seniority presence and process (e.g., high RSI checklist usage). In contrast to this to the recent study by Russotto et al.^25^ of nearly 3000 critically ill patients demonstrated cardiovascular instability in 42% of their cohort and a first‐pass success rate of only 80%, although the majority of their patients were treated in the intensive care unit rather than the ED and so may represent a different population. The high first‐pass success rate in our study is also in contrast to a study by Sivilotti et al.^26^ where different success rates with alternative sedation regimes were seen during RSI, although the paralytic medications and doses employed varied and were generally significantly lower than seen in our study. The relatively high dose of rocuronium used (1.5 mg/kg), ubiquity of videolaryngoscopy, and standardized approach are likely to explain the similarly high rates of intubation success seen in both arms of our study rather than these being due to differences in sedative choice.

Rates of hypoxia were similar between groups, which may reflect the relatively modest doses of fentanyl administered and also the fact that the medications were administered in a rapid sequence fashion rather than the fentanyl (or placebo) being given as a pretreatment. Because the divergence of blood pressures seen between the two groups was already established by the 2‐min time point, clinicians who may be concerned that fentanyl needs to be predosed with a longer interval prior to induction should be reassured that this did not appear to be the case.

The participant group studied was heterogenous. While on the one hand this is a strength, improving the external validity of our findings, it is likely that some pathologies require different hemodynamic targets for optimal outcomes (e.g., in traumatic brain injury the avoidance of hypotension may be imperative, whereas in aneurysmal subarachnoid hemorrhage, avoiding hypertension may be key). Due to relatively small numbers of any given pathology, our study is unable to inform these more granular questions with any accuracy.

While further study is needed to clarify an optimal regimen in different circumstances, our data provide clinicians with more information on which to judge their induction medication choices, taking into account their understanding of the patient's presumed pathology and baseline hemodynamics. Given our results it would be reasonable to omit fentanyl in patients where hypotension is judged to be detrimental and to include it in the induction regimen for patients with hypertension at baseline. For those patients receiving fentanyl at the time of induction, the ideal dose in different circumstances requires further clarification.

## LIMITATIONS

As a result of our binary end point there will be patients at the margins where classification into either category is unlikely to be clinically significant. Selection bias may have arisen as a result of our inability to reliably recruit overnight due to staffing limitations, and reliance on noninvasive blood pressures may have resulted in some measurement error. Our protocol discouraged use of additional sedation during the 10 min following induction to avoid confounding; in practice, without this constraint, clinicians may be able to manage hypertension by means of titration of sedative medications. Finally, our study was not powered to detect differences in mortality.

## CONCLUSIONS

While there was no difference between the proportion of patients in each group who met the primary outcome of a blood pressure measurement falling outside the target range within the 10 min following induction, systolic blood pressures were significantly lower in the fentanyl group. No difference was seen in intubating conditions, mortality, or duration of mechanical ventilation. We suggest that clinicians take into account baseline hemodynamics and a targeted postinduction blood pressure when deciding whether to add fentanyl to an induction regimen of ketamine and rocuronium.

## CONFLICT OF INTEREST

The authors have no potential conflicts to disclose.

## AUTHOR CONTRIBUTIONS

Ian Ferguson, Brian Burns, and Cliff Reid conceived the study. Ian Ferguson, Brian Burns, Cliff Reid, Alexander Buttfield, Shamus Shepherd, James Milligan, and Anders Aneman designed the trial, and Ian Ferguson obtained ethical approval and trial registration. Ian Ferguson, Brian Burns, Cliff Reid, Alexander Buttfield, Shamus Shepherd, and James Milligan undertook recruitment at participating centers and managed the data, including quality control. Anders Aneman and Ian A. Harris provided statistical advice and methodological support. Ian Ferguson drafted the manuscript and all authors contributed to revision and approval of the final version. Ian Ferguson takes responsibility for the paper as a whole.

## Supporting information


Table S1
Table S2
