# Supplementary material for: Fentanyl versus placebo with ketamine and rocuronium for patients undergoing rapid sequence intubation in the emergency department: The FAKT study—A randomized clinical trial
Source: Acad Emerg Med. 2022 Mar 15;29(6):719–28. doi: 10.1111/acem.14446 (PMC9314707; doi:10.1111/acem.14446)
Supplement: Supplementary file 1 — Table S1 Table S2 [file ACEM-29-719-s001.docx]

**Online Supplement**

**Table 1. Inclusion and exclusion criteria**

| **Inclusion criteria** | **Exclusion criteria** |
| --- | --- |
| Adult patient (≥18 years) | Allergic to study medications |
| Requires intubation in the emergency department | Paralysis only or ‘cold’ intubation. |
|  | Alternative induction regimen needed in the judgement of the treating emergency physician. |
|  | No emergency physician trained in the protocol available. |
|  | Overwhelmed emergency department. |
|  | Other |

**Table 2. Weight based dosing schedule.**

|  | **Standard Dose** | | | **Reduced Dose** | | |
| --- | --- | --- | --- | --- | --- | --- |
| **Weight** | **Study drug** | **Ketamine** | **Rocuronium** | **Study drug** | **Ketamine** | **Rocuronium** |
| **40-50kg** | 6-8ml | 60-80mg | 60mg | 2-4ml | 20-40mg | 60mg |
| **51-60kg** | 7.5-10ml | 75-100mg | 75mg | 2.5-5ml | 25-50mg | 75mg |
| **61-70kg** | 8-12ml | 80-120mg | 90mg | 3-6ml | 30-60mg | 90mg |
| **71-80kg** | 100-14ml | 100-140mg | 100mg | 3.5-7ml | 35-70mg | 100mg |
| **81-90kg** | 12-16ml | 120-160mg | 120mg | 4-8ml | 40-80mg | 120mg |
| **91-100kg** | 14-18ml | 140-180mg | 140mg | 4.5-9ml | 45-90mg | 140mg |
| **101kg+** | 15-20ml | 150-200mg | 150mg | 5-10ml | 50-100mg | 150mg |
